# 2-Hydroxy-Docosahexaenoic Acid Is Converted Into Heneicosapentaenoic Acid via α-Oxidation: Implications for Alzheimer’s Disease Therapy

**DOI:** 10.3389/fcell.2020.00164

**Published:** 2020-03-27

**Authors:** Sebastià Parets, Ángel Irigoyen, Margarita Ordinas, Joan Cabot, Marc Miralles, Laura Arbona, Mária Péter, Gábor Balogh, Paula Fernández-García, Xavier Busquets, Victoria Lladó, Pablo V. Escribá, Manuel Torres

**Affiliations:** ^1^Laboratory of Molecular Cell Biomedicine, Department of Biology, University of the Balearic Islands, Palma de Mallorca, Spain; ^2^Department of Neurosciences and Neurology, Laminar Pharmaceuticals SL, Palma de Mallorca, Spain; ^3^Instrumental Techniques Laboratory, DDUNAV-Drug Development Unit-University of Navarra, Pamplona, Spain; ^4^Institute of Biochemistry, Biological Research Centre, Szeged, Hungary

**Keywords:** omega-3 polyunsaturated fatty acids, α-oxidation, Alzheimer’s disease, DHA, hydroxyl derivatives, lipid metabolism, membrane lipid therapy

## Abstract

Alzheimer’s disease (AD) is a neurodegenerative disease with as yet no efficient therapies, the pathophysiology of which is still largely unclear. Many drugs and therapies have been designed and developed in the past decade to stop or slow down this neurodegenerative process, although none has successfully terminated a phase-III clinical trial in humans. Most therapies have been inspired by the amyloid cascade hypothesis, which has more recently come under question due to the almost complete failure of clinical trials of anti-amyloid/tau therapies to date. To shift the perspective for the design of new AD therapies, membrane lipid therapy has been tested, which assumes that brain lipid alterations lie upstream in the pathophysiology of AD. A hydroxylated derivative of docosahexaenoic acid was used, 2-hydroxy-docosahexaenoic acid (DHA-H), which has been tested in a number of animal models and has shown efficacy against hallmarks of AD pathology. Here, for the first time, DHA-H is shown to undergo α-oxidation to generate the heneicosapentaenoic acid (HPA, C21:5, n-3) metabolite, an odd-chain omega-3 polyunsaturated fatty acid that accumulates in cell cultures, mouse blood plasma and brain tissue upon DHA-H treatment, reaching higher concentrations than those of DHA-H itself. Interestingly, DHA-H does not share metabolic routes with its natural analog DHA (C22:6, n-3) but rather, DHA-H and DHA accumulate distinctly, both having different effects on cell fatty acid composition. This is partly explained because DHA-H α-hydroxyl group provokes steric hindrance on fatty acid carbon 1, which in turn leads to diminished incorporation into cell lipids and accumulation as free fatty acid in cell membranes. Finally, DHA-H administration to mice elevated the brain HPA levels, which was directly and positively correlated with cognitive spatial scores in AD mice, apparently in the absence of DHA-H and without any significant change in brain DHA levels. Thus, the evidence presented in this work suggest that the metabolic conversion of DHA-H into HPA could represent a key event in the therapeutic effects of DHA-H against AD.

## Introduction

Alzheimer’s disease (AD) is a devastating neurodegenerative disease that remains an unmet therapeutic need despite the tremendous investments made in basic and clinical research into AD. The absence of efficient therapeutic approaches for this pathology must be, at least in part, attributed to the limited knowledge of the key molecular events leading to neurodegeneration. The Disease Modifying Drugs (DMDs) designed and tested in clinical trials have reported adverse effects and/or no clinical improvement. Most of these DMDs are drugs or biological agents (immunotherapy) designed to inhibit or modulate the amyloid pathology and/or tauopathy evident in AD (Torres et al., 2016). Both these neuropathological features have long been considered to be the molecular events triggering AD-related pathophysiological events, in accordance with the amyloid cascade hypothesis (Selkoe and Hardy, 2016). However, this concept is challenged by the fiascos of clinical trials with anti-amyloid/tau therapies to date (Krstic and Knuesel, 2013; Herrup, 2015; Torres et al., 2016; Ricciarelli and Fedele, 2017). In this context, AD has been associated with several lipid alterations that are related to neurodegeneration, such as hypercholesterolemia (Martins et al., 2009), alterations in sphingomyelin storage (Tamboli et al., 2011), and a downregulation of docosahexaenoic acid (DHA) and related bioactive hydroxylated mediators like NPD1 (Neuroprotectin D1) (Prasad et al., 1998; Lukiw et al., 2005; Astarita et al., 2010). DHA is an omega-3 polyunsaturated fatty acid (PUFA) that is the most abundant lipid in the human brain and that is required for normal cognitive development (Lim et al., 2005; Yamashima, 2012; Torres et al., 2016). Apart from DHA, other omega-3 PUFAs are also diminished in the brains of AD patients (e.g., EPA, eicosapentaenoic acid), usually esterified to phosphatidylethanolamine (PE) (Chan et al., 2012). This decrease in the levels of DHA and of other omega-3 PUFAs could constitute the basis for the onset of the pathology and consequently, they could represent a suitable therapeutic target.

There is evidence from cell and animal models that therapies based on nutritional supplement with DHA have neuroprotective effects (Morris et al., 2003; Su, 2010; Dyall, 2015; Hennebelle et al., 2015; Belkouch et al., 2016). However, administration of DHA and/or EPA to AD patients in clinical trials has only been effective in small groups of patients with a mild-to-moderate pathology (Freund-Levi et al., 2006; Quinn et al., 2010). Here, a membrane lipid therapy perspective is adopted for AD, on the assumption that the main events triggering AD pathophysiology are related to membrane lipid composition and structure (Escribá et al., 2008, 2015). DHA-H (2-hydroxy-docosahexaenoic acid) is an MLT-based molecule, designed as a therapeutic alternative to combat neurodegenerative disorders like AD. Results from cell and animal models support the potential of DHA-H to treat AD. Data from a transgenic model of AD (5xFAD) has shown that DHA-H prevents cognitive decline in animals that received early treatment (from 3 to 7 months of age) (Fiol-Deroque et al., 2013; Mohaibes et al., 2017). In this sense, this effect on cognitive capacity is accompanied by an increase in neuronal cell proliferation in the hippocampus, suggesting that this therapy is mediated, at least in part, by a recovery of neurogenesis to healthy control levels (Fiol-Deroque et al., 2013). In addition, the neuropathological features associated with AD, such as tau protein phosphorylation and β-amyloid peptide accumulation (microscopic lesions characteristic of AD), are significantly dampened in the brain of 5xFAD animals treated with DHA-H (Torres et al., 2014). Hence, the use of DHA-H appears likely to preserve neuronal density, as seen in cell models where DHA-H prevents the neuronal death induced by β-amyloid oligomers or by NMDA (N-Methyl-D-Aspartate) and calcium (excitotoxicity) (Torres et al., 2014, 2015). Finally, DHA-H also has beneficial effects in an animal model of Parkinson’s disease, where it prevented oxidative stress and dopaminergic neuron death (Hernando et al., 2019).

Here, we characterized several aspects of DHA-H metabolism toward HPA (heneicosapentaenoic acid), also measuring the levels of DHA-H and HPA in plasma and in the brain of WT and 5xFAD mice to establish a link between DHA-H metabolism and its therapeutic effects. The enzymatic conversion of DHA-H into HPA was first characterized in cell models and its effects on the cellular fatty acid composition were assessed and compared with those of the native DHA. In addition, the key events that provoke the differences in cell lipid composition following treatment with DHA-H and DHA are also addressed. Finally, the DHA-H and HPA levels were also determined in mice treated with DHA-H and DHA. Brain levels of HPA were positively correlated with spatial behavior in 5xFAD mice, with virtually no DHA-H detected and the DHA levels unmodified in the brain.

## Materials and Methods

### Lipids, Reagents, and Organic Solvents

DHA (docosahexaenoic acid sodium salt; C22:6 n-3) and DHA-H (2-hydroxy-docosahexaenoic acid sodium salt; 2OH-C22:6 n-3) were obtained from Laminar Pharmaceuticals (Spain). Margaric acid (free fatty acid; C17:0) was purchased from Sigma-Aldrich and HPA (heneicosapentaenoic acid as a free fatty acid; C21:5 n-3) from Cayman Chemicals (Michigan, United States). D(+)-Glucose (cell culture tested), sodium pyruvate, L-Gln (cell culture tested), acetyl chloride and N,O-bis (trimethylsilyl) acetamide, sodium chloride, sodium phosphate, EDTA (Ethylenediaminetetraacetic acid) and tris-base were all obtained from Sigma-Aldrich. By contrast, HPLC grade chloroform, ethanol, methanol, hydrochloric acid, and hexane were all obtained from Scharlab (Spain). For lipidomic analysis, methanol, chloroform and isopropanol were all LC-MS grade from Thermo Scientific or Sigma-Aldrich. In addition, butylated hydroxytoluene, dimethylformamide, ammonium chloride were all the best available grade from Sigma-Aldrich. Lipidomic standards were obtained from Avanti Polar Lipids (Alabaster, AL, United States) and Nu-Chek-Prep (Elysian, MN, United States). Heparin (5000 units/mL) was purchased from Hospira Invicta S.A. (Spain), Ketamine (Anesketin 100 mg/mL) from Eurovet Animal Health BV (The Netherlands), Xilacine (Xilagesic 20 mg/mL) from Laboratorios Calier S.A. (Spain), and oxythiamine hydrochloride from Santa Cruz Biotechnology (Germany).

### Cell Culture and Treatments

HEK293T cells were cultured in Dulbecco’s Modified Eagle Medium (DMEM: Biowest, France) supplemented with 10% FBS (Fetal Bovine Serum; Gibco, Thermo Fisher Scientific), 2 mM L-Gln, 25 mM D(+)-glucose, 1mM sodium pyruvate and penicillin/streptomycin. Mouse neuroblastoma N2a cells were maintained in a 1:1 (v:v) mixture of DMEM and Opti-Mem (Gibco, Thermo Fisher Scientific), supplemented with 5% FBS and penicillin/streptomycin. Both cell lines were incubated in an atmosphere of 5% CO_2_ at 37°C.

Cells were plated at different densities in accordance with the time of incubation with the drug. HEK293T cells were plated in 90-mm-diameter dishes at 4 × 10^6^ cells/plate for a 6 h incubation, 2 × 10^6^ for a 24 h incubation, 1 × 10^6^ for 48 h incubation and 5 × 10^5^ for 72 h incubation. N2a cells were plated in 90 mm-diameter dishes at 2.5 × 10^6^ cells/plate for a 6 h incubation, 1.25 × 10^6^ for a 24 h incubation, 6 × 10^5^ for 48 h incubation and 4 × 10^5^ for a 72 h incubation. All cultured plates reached 90% confluence by the time of collection. HEK293T cells were incubated with DHA-H and DHA at 10, 30, and 100 μM for 24 h, and at 30 μM for 6, 48, and 72 h. N2a cells were incubated with DHA-H at 30 μM for 6, 24, 48, and 72 h. In addition, HEK293T cells were also incubated with oxythiamine (water soluble) in the presence of DHA-H under the same conditions, at a final oxythiamine concentrations of 1 and 10 mM. HEK293T cells were detached from the plates by pipetting with cold PBS (Phosphate Buffer Saline), whereas N2a cells were collected by trypsinization (3–5 min, 37°C). The cells were recovered by centrifugation (1000 × g, 10 min at 4°C) and washed twice with cold PBS before they were frozen at −80°C.

To analyze the levels of DHA-H and DHA in the cell culture medium, 90 mm diameter dishes were filled with 11 mL of complete cell culture medium containing 30 μM DHA-H or DHA in the presence or absence of plated HEK293T cells (5 × 10^5^ cells/plate). The plates were incubated as described above and 1 mL aliquots were collected from the plates at 0, 6, 24, 48, and 72 h. Aliquots of the cell’s culture medium were immediately centrifuged at 1000 × g for 10 min at 4°C in order to remove any cells in suspension and the cell-free aliquots were stored at −20°C.

### Animals, Treatments, and Sample Collection

For the analysis of DHA-H and HPA blood plasma levels, 6-month-old wild type mice (B6/SJL hybrid genetic background) each weighing 30–35 g were orally administered DHA-H (200 mg/kg.day) for 14 days. The pharmacokinetic (PK) profile of DHA-H was determined on day 15 by blood withdrawal 1, 2, 3, 4, 6, 8, and 24 h post-administration. The blood sample for time 0 (basal levels before drug administration) was collected on day 14 rather than day 15 in order to avoid additional animal stress. The average total blood volume per mouse is about 80 mL/kg (0.080 mL/g) (Evans, 1994)^1^, so that for a mouse weighing 30–35 g, the total blood volume should be around 2.4 and 2.8 mL. Blood was obtained by submandibular bleeding (Golde et al., 2005) and the volume withdrawn at each time point was approximately 40 μL (2 blood drops), so the nominal blood volume removed on day 15 was 0.28 mL. This protocol was designed to meet the criteria approved by the Bioethical Committee at the University of the Balearic Islands for this procedure, which established that the total volume withdrawn during one experimental session should be no higher than 10% of the total blood volume. Blood was collected in heparinized tubes and centrifuged at room temperature for 10 min at 1500 × g, in order to separate platelet-free plasma from the cell pellets. The volume of plasma collected was quantified and frozen at −80°C until analysis. This PK study was performed on three different mice.

The 5xFAD model of Alzheimer’s disease is a double transgenic PS1/APP mouse that harbors five human mutations associated with familial AD (line Tg6799): the Swedish (K670N/M671L), Florida (I716V), and London (V717I) mutations in APP; and the M146L and L286V clinical mutations in human PS1. Both transgenes are expressed under the control of the Thy-1 promoter and the mice display cognitive decline from 4 months of age (Oakley et al., 2006). Transgenic 5xFAD and wild type (WT) mice were obtained from Jackson Laboratories (United States) and they were maintained on a B6/SJL genetic background by crossing heterozygous transgenic mice with B6/SJL WT (F1) breeders. All animals were housed under controlled temperature of 22°C (± 2°C) and 70% humidity, on a 12 h–12 h light-dark cycle, with *ad libitum* access to a standard laboratory diet (Panlab A03, Barcelona, Spain).

WT and 5xFAD transgenic male mice received DHA-H (or DHA) orally, dissolved in 5% ethanol, at a daily dose of 5, 20, 50, and 200 mg/kg, or the vehicle solution alone. These treatments started when the mice reached 3 months of age (dosed 5 days/week) and they were continued until the mice reached 7 months of age. During the last month of treatment, all the animals were maintained on a hypocaloric diet to perform the selected behavioral spatial learning and memory tests (food craving test in a radial arm maze, see below). A total of 46 animals were used for this study: vehicle-treated WT (*n* = 3), DHA-H-treated WT (20 mg/kg, *n* = 3; and 200 mg/kg, *n* = 3), DHA-treated WT (20 mg/kg, *n* = 3); vehicle-treated 5xFAD (*n* = 5), DHA-H-treated 5xFAD (5 mg/kg, *n* = 6; 20 mg/kg, *n* = 5; 50 mg/kg, *n* = 6; and 200 mg/kg, *n* = 7), and DHA-treated 5xFAD (20 mg/kg, *n* = 5) mice. Following the behavioral test, the mice were maintained on a normal diet (and treatment) for 1 week more, after which they were anesthetized with an intraperitoneal injection of ketamine/xylazine (100/10 mg/kg) and perfused intracardially with 50 mL of heparinized saline solution. The animal’s brain was then removed immediately and dissected down the midline on a cold surface. Having removed the cerebellum, each cerebellum-free hemibrain was frozen in liquid nitrogen and stored at −80°C. All the protocols employed were approved by the Bioethical Committee at the University of the Balearic Islands, and they are in accordance with national and international guidelines on animal welfare.

### Radial Arm Maze (RAM) Test

The spatial behavior test was performed as described previously, with some modifications (Fiol-Deroque et al., 2013). All the animals were isolated and submitted to food restriction until reaching 80–85% of the normal body weight in *ad libitum* feeding, and they were maintained in these conditions just 1 week before starting the trial and until the end of the test. After food restriction and 3 days before trials started, the animals were trained twice daily in the eight radial arm maze (RAM) test (LE766/8, Panlab SL, Spain) over the 3 days. Each mouse was placed in the middle of the maze and allowed to look for the reward, a 45 mg food pellet (Dustless Precision Pellets, Bio-Serv, United States), located at the end of every arm. Each session finished when the animal either succeeded in finding the eight baited arms or failed in complete all the arms in 10 min. The movement of each animal was recorded with a digital video tracking system (LE 8300 with software Sedacom v1.3, Panlab, SL, Spain) and after the training, the experimental paradigm started. In all the experimental sessions (1 session per day), just four arms were baited compared to the training protocol, and each session finished when the animals either succeeded in finding the four baited arms or failed after 10 min. Performance was assessed taking into account: (1) the time to achieve the test; (2) the number of Working Memory Errors (WMEs, re-entry into a previous visited baited arm); (3) the number of Reference Memory Errors (RMEs, entry into an unbaited arm); and (4) the total number of errors (WME+RME). The test was repeated 5 days/week for 3 weeks. Once the RAM test was finished, animals were fed *ad libitum* for one extra week before sacrifice.

### Lipid Extraction and Fatty Acid Transmethylation

Cell pellets from HEK293T or N2a cells were lysed in cold hypotonic buffer (1 mM EDTA, 20 mM Tris-HCl [pH 7.4]) by pipetting up and down. The cell lysates were subjected to ultrasound pulses (4 cycles, 10 s/cycle, 10W) before lipid extraction. For hemi-brain analysis, the tissue from each animal was homogenized in cold PBS at 1:10 (w:v) in the presence of protease inhibitors (Roche), using a blade homogenizer (Polytron PT3100). Homogenates were further subjected to ultrasound, and then aliquoted and stored at −80°C. Just one aliquot from each sample, containing about 8 mg of protein/aliquot, was subjected to lipid extraction. The protein content prior to lipid extraction was determined by a modified Lowry’s method (Bio-Rad DC Protein Assay).

Margaric acid (C17:0) was added to the samples undergoing lipid extraction as an internal standard and the lipids were extracted by chloroform:methanol extraction (Eggers and Schwudke, 2016). Briefly, 0.75 volumes of the aqueous phase (already containing the biological sample) were mixed with 2 volumes of chloroform and 1 volume of methanol. This mixture was vortexed for 1 min and centrifuged at 1000 × g for 10 min. The lower organic phase was collected and washed with 1 mL of PBS:methanol (1:1, v:v), and the resultant organic phase was dried under argon flow. The film containing the extracted lipids was transmethylated by incubating the lipid mixture for 90 min at 100°C in 3 mL methanol:acetyl chloride (10:1, v:v), under an argon atmosphere and in pyrex screw-capped tubes (Christie, 1993). The resultant fatty acid methyl esters (FAMEs) were extracted with hexane, adding 3 mL H_2_O and 1 mL hexane to the transmethylation reaction, and thoroughly vortexing the mixture. After centrifugation at room temperature (1,000 × g for 10 min), the upper phase containing the FAMEs was collected and the remaining volume was washed twice with 1 mL hexane. The hexane phases were combined, evaporated under argon flow and resuspended in 60 μL of hexane (for cell, cell culture medium and blood plasma sample analysis) or in 200 μL (for brain sample analysis). In order to check if a fatty acid compound is hydroxylated, isolated FAMEs were subjected to a second derivatization with trimethylsilyl (Alderson et al., 2004). Briefly, FAMEs were dried under argon flow and the lipid film was dissolved in N,O-bis (trimethylsilyl) acetamide (0.1–5.0 mg lipid for 200–400 μL trimethylsilylation reagent), which was in turn heated in a capped vial at 70°C for 30 min. The solvent was evaporated and the lipid film was resuspended in hexane for analysis. When the fatty acid under study was hydroxylated, the retention time of the related FAME moved as a result of this second derivatization. However, if the fatty acid under study was not hydroxylated, the resultant FAME displayed the same retention time irrespective of the second derivatization.

### Gas Chromatography Coupled to Flame Ionization Detector

Fatty acids were subjected to methylation or to methylation/trimethylsilylation, and they were analyzed on an Agilent 7890A GC system equipped with a Flame Ionization Detector (GC-FID) and a 7693 auto-injector (Agilent Technologies, CA, United States). An Agilent J&W HP-5MS capillary column (30 m × 0.25 mm i.d.) coated with a 0.25 μm stationary phase (5% phenyl–methylsiloxane) was used, with 1.2 mL/min of helium as a carrier. For GC separation, the column was equilibrated at 180°C for 5 min upon sample injection (1 μL at a split ratio of 5:1). The temperature was raised at a rate of 2°C/min up to 230°C, and then to 280°C at a rate of 5°C/min. The injector and detector temperatures were maintained at 250 and 290°C, respectively. The areas under the peaks were quantified using margaric acid (C17:0) as the internal standard and the results were normalized accordingly to the sample’s protein content. Peaks were identified using standards for the different hydroxylated and non-hydroxylated fatty acids. A linear correlation was observed between the peak area (FID Signal) and the concentration of fatty acid injected, while the fatty acid standards under study had very similar slopes (peak area vs. concentration) and similar to the internal standard (Supplementary Figure S1).

### Gas Chromatography Coupled to Mass Spectrometry

Chromatographic analyses were carried out on an Agilent 6890 gas chromatography apparatus coupled to an Agilent 5973 quadrupole mass spectrometer (GC-MS). The apparatus was equipped with an HP-5MS capillary column (30 m × 0.25 mm i.d.) coated with a 0.25 μm film of the stationary phase (5% phenyl–methylsiloxane) and GC was performed as descried above for GC-FID, with high purity helium as the carrier gas flowing at a rate of 1.2 mL/min, injecting 1 μL in Splitless mode. The injector was held at 250°C and the transfer line to the detector at 290°C. The GC oven temperature was programmed at 180°C and it increased at a rate of 2°C/min up to 230 C, and then at 5°C/min to 280°C. The mass spectrometer was operated in electron impact mode (EI, 70 eV), and in scan mode from 50 to 550 uma.

### Lipidomic Analysis

Calculated amount of methanol (500 μL/mg of protein) containing 0.001% butylated hydroxytoluene (BHT) and calculated amount of PC(40:0) (10 μg/mg of protein) as extraction standard were added to the frozen cell pellets. Samples were sonicated for 5 min in bath sonicator (in ice-cold water), then shaken for 5 min, and centrifuged at 10,000 × g for 5 min. The supernatant was transferred into a fresh tube and stored at −20°C until MS measurement.

Electrospray ionization (ESI)-MS analyses were performed on a high-sensitivity, high-resolution Orbitrap Fusion Lumos instrument (Thermo Fisher Scientific) equipped with a TriVersa NanoMate robotic nanoflow ion source (Advion BioSciences), using chips with 5.5 μm diameter spraying nozzles. The instrument was fully calibrated before analysis. The ion source was controlled by Chipsoft 8.3.1 software. Ionization voltages were +1.3 and -1.9 kV in positive and negative modes, respectively, and back-pressure 1 psi for both modes. The temperature of the ion transfer capillary was 330°C. Acquisitions were performed at the mass resolution R_m/z=__200_ = 240,000. 3 μL lipid extract was diluted with 150 μL infusion solvent mixture (chloroform:methanol:iso-propanol 1:2:1, by vol.) containing an internal standard mix (71 pmol PC D31-16:0/18:1, 5 pmol SM d18:1/17:0, 5 pmol DG 15:0-D7-15:0, 19 pmol TG 15:0-18:1-D7-15:0, 23 pmol 18:1 Chol (D7) ester, 25 pmol PE D31-16:0/18:1, 11 pmol PI D31-16:0/18:1, 19 pmol PS D31-16:0/18:1, 2.5 pmol PG D31-16:0/18:1, 1 pmol PA D31-16:0/18:1, 1.5 pmol CL 56:0, 2 pmol Cer d18:1/17:0, 3 pmol GluCer d18:1/12:0, and 5 pmol GM3 D3-d18:1/18:0, and 10 pmol FFA 19:0). Next, the mixture was halved, and 5% dimethylformamide (additive for the negative ion mode) or 3 mM ammonium chloride (additive for the positive ion mode) were added to the split sample halves. 10 μL solution was infused and data were aquired for 2 min.

Phosphatidylcholine (PC, diacyl and PC-O, alkyl-acyl), lysophosphatidylcholine (LPC), sphingomyelin (SM), diacylglycerol (DG), triacylglcerol (TG) and cholesteryl ester (CE) were detected and quantified using the positive ion mode, while phosphatidylethanolamine (PE, diacyl and PE-P, alkenyl-acyl), lysophosphatidylethanolamine (LPE), phosphatidylinositol (PI), lysophosphatidylinositol (LPI), phosphatidylserine (PS), lysophosphatidylserine (LPS), phosphatidic acid (PA), lysophosphatidic acid (LPA), phosphatidylglycerol (PG), lysophosphatidylglycerol (LPG), cardiolipin (CL), ceramide (Cer), hexosyl ceramide (HexCer), ganglioside GM3 and free fatty acids were detected and quantified using the negative ion mode.

Data-dependent MS2 and MS3 fragmentation experiments were performed to confirm the structures of lipid species containing DHA-H or HPA as fatty acyls. Normalized HCD collision energies were ramped from 20 to 35 in steps of 5.

Raw MS spectra were converted to platform-independent mzML files, and lipid species were identified using the LipidXplorer software (Herzog et al., 2011). Identification was made by matching the m/z values of their monoisotopic peaks to the corresponding elemental composition constraints. The mass tolerance was set to 3 ppm. Data files generated by LipidXplorer queries were further processed by in-house Excel macros.

### Statistical Analysis

The data were expressed as the mean ± SEM and to compare between several groups, we used one-way ANOVA followed by a Tukey’s *post hoc* multiple comparison (GraphPad Prism 6.1). The level of significance was set at 95% confidence (*p* < 0.05). Linear and polynomial inverse regressions were adjusted to the individual experimental points (Sigma Plot 9.0), and each regression shown converged in 1 iteration with the tolerance satisfied. The adjusted r^2^ parameter and *p*-value are shown for each regression.

## Results

### Heneicosapentaenoic Acid (HPA) Is Produced in DHA-H-Treated HEK293T Cells via α-Oxidation

**FIGURE 1:**
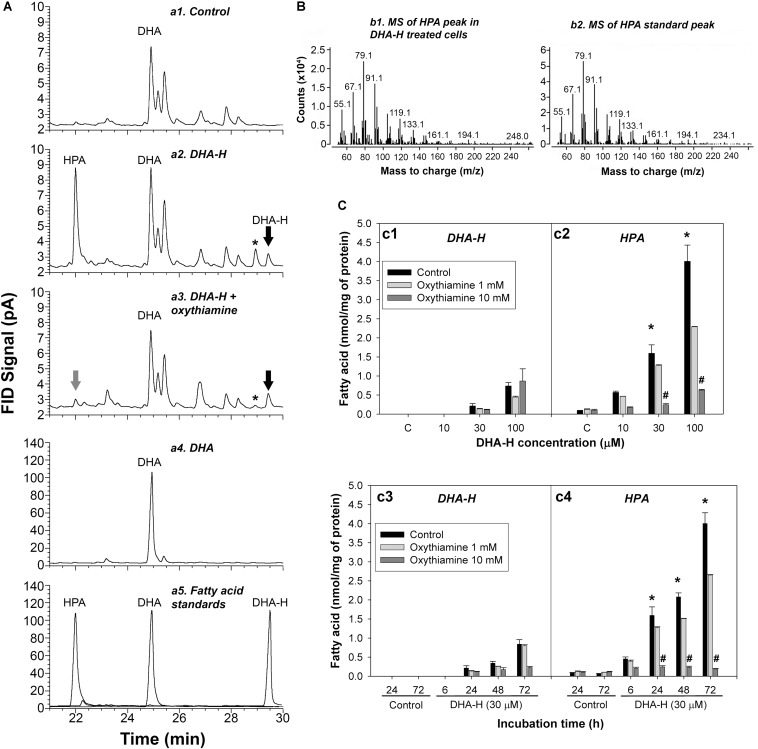
DHA-H is converted into HPA in HEK293T cells via α-oxidation. **(A)** Amplified regions of representative GC-FID chromatograms showing the fatty acid composition under different experimental conditions: control (a1); DHA-H treatment (100 μM, 24 h – a2); DHA-H treatment (100 μM, 24 h) plus oxythiamine (10 mM, 24 h – a3); DHA (100 μM, 24 h – a4); and fatty acid standards (a5). Peaks of interest were identified according to their retention time, with the peak marked with an asterisk modulated by DHA-H (and oxythiamine) but with an unknown molecular identity. **(B)** Mass spectra for the HPA peak in DHA-H-treated cells (100 μM, 24 h) and of the HPA standard. Both spectra displayed main fragments with an identical mass-to-charge ratio (m/z). **(C)** Intracellular levels of DHA-H (c1) and HPA (c2) in cells treated with increasing concentrations of DHA-H for 24 h and intracellular levels of DHA-H (c3) and HPA (c4) at different times of incubation (DHA-H, 30 μM), in the presence or absence of oxythiamine to inhibit α-oxidation. Both DHA-H and HPA increased in a concentration- and time-dependent manner, with significantly higher HPA levels than DHA-H on exposure to 30 μM DHA-H from 24 h onward. This increase in HPA was not evident in the presence of 10 mM oxythiamine. Bars represent mean ± SEM, and the statistical analysis was performed by one-way ANOVA and with Tukey’s *post hoc* test: *p < 0.05 when comparing HPA vs. DHA-H levels; ^#^*p* < 0.05 when comparing values in the presence and absence of 10 mM oxythiamine.

HEK293T cells in culture were exposed to distinct concentrations of DHA-H for different times and the modification to the cell’s fatty acid profile was evaluated. The fatty acid profile of these cells was determined by GC-FID (see Figure 1A) and the most remarkable effect was the appearance of 3 peaks with retention times ranging between 21 and 30 min upon exposure to DHA-H (compare panels a1 and a2). One of these peaks was identified as the DHA-H added to the culture (see the black arrow in panel a2), whereas the other two peaks were not immediately identified. In accordance with its retention time, the most abundant peak appearing after DHA-H stimulation (see chromatogram in panel a2 at 22 min) was recognized as an odd-chain non-hydroxylated fatty acid containing 21 carbons. The fatty acid hydroxylation status of this peak was tested by trimethylsilylation of the FAME, which did not change its retention time, demonstrating the absence of hydroxyl groups. Interestingly, this peak had the same retention time as a commercially available C21:5 n-3 standard (HPA, compare panels a2 and a5). To confirm the molecular identity of this peak, the peak observed after DHA-H treatment and the HPA peak corresponding to the commercial standard were analyzed by GC-MS. The molecular fragments observed in the mass spectra from both these molecules were identical in the mass-to-charge (m/z) ratios, indicating that the peak that appeared after DHA-H treatment corresponded to HPA (Figure 1B). Nevertheless, a final peak identified after exposure of the cell to DHA-H treatment remained unidentified (see panel a2, the peak labeled with an asterisk). Similar results were also obtained from mouse neuroblastoma cells (N2a) following exposure to DHA-H (Supplementary Figure S2).

The production of HPA following the exposure of 293T cells to DHA-H merited further attention. HPA accumulated in the cells exposed to DHA-H in a concentration- and time-dependent manner (Figure 1C, panels c1–c4). After stimulation with growing concentrations of DHA-H for 24 h, the levels of both DHA-H and HPA were higher than those in the control cells, with significantly higher levels of HPA than those of DHA-H itself at 30 and 100 μM (Figure 1C, panels c1-c2, black bars). Likewise, exposure to a fixed concentration of 30 μM DHA-H also led to the accumulation of HPA and DHA-H over time, with significantly higher levels of HPA than those of DHA-H itself from 24 h onward (Figure 1, panels c3-c4, see black bars). In this sense, it is known that saturated 2-hydroxy fatty acids can be converted into their corresponding odd-chain fatty acid containing n-1 carbon atoms. Such metabolic pathways involve peroxisomal α-oxidation of the 2-hydroxy fatty acid, and 1 carbon is lost from the carboxyl group to generate a non-hydroxylated fatty acid containing n-1 carbon atoms. Interestingly, this metabolic pathway has not been yet described for long chain PUFAs like DHA-H. To demonstrate that this pathway mediates DHA-H metabolism, cells were treated with the competitive antagonist of thiamine pyrophosphate (TPP), oxythiamine, which in turn inhibits 2-hydroxyphytanoyl-CoA lyase (2-hydroxyacyl-CoA lyase 1; HACL1), the main enzyme involved in fatty acid α-oxidation (Foulon et al., 2005). The increase in HPA in cells exposed to DHA-H was almost completely abolished in the presence of 10 mM oxythiamine (see Figure 1A, panels a2 and a3), and this inhibitory effect of oxythiamine on HPA production was concentration dependent (Figure 1C, panels c2 and c4, gray bars). Hence, HACL1 appears to be implicated in DHA-H metabolism to HPA, yet interestingly, the inhibitory effect of oxythiamine was not accompanied by the accumulation of the substrate, DHA-H (see Figure 1C, panels c1 and c3, gray bars). Since the enzymatic activity of HACL1 depends on prior activation of the substrate with coenzyme A (CoA), most of DHA-H must be conjugated to CoA after oxythiamine inhibition. Since CoA is highly hydrophilic, DHA-H-CoA would be preferentially lost in the lipid extraction performed before GC analysis, which would explain the failure to accumulate this substrate after oxythiamine inhibition in these assays (Olbrich et al., 1981).

**FIGURE 2:**
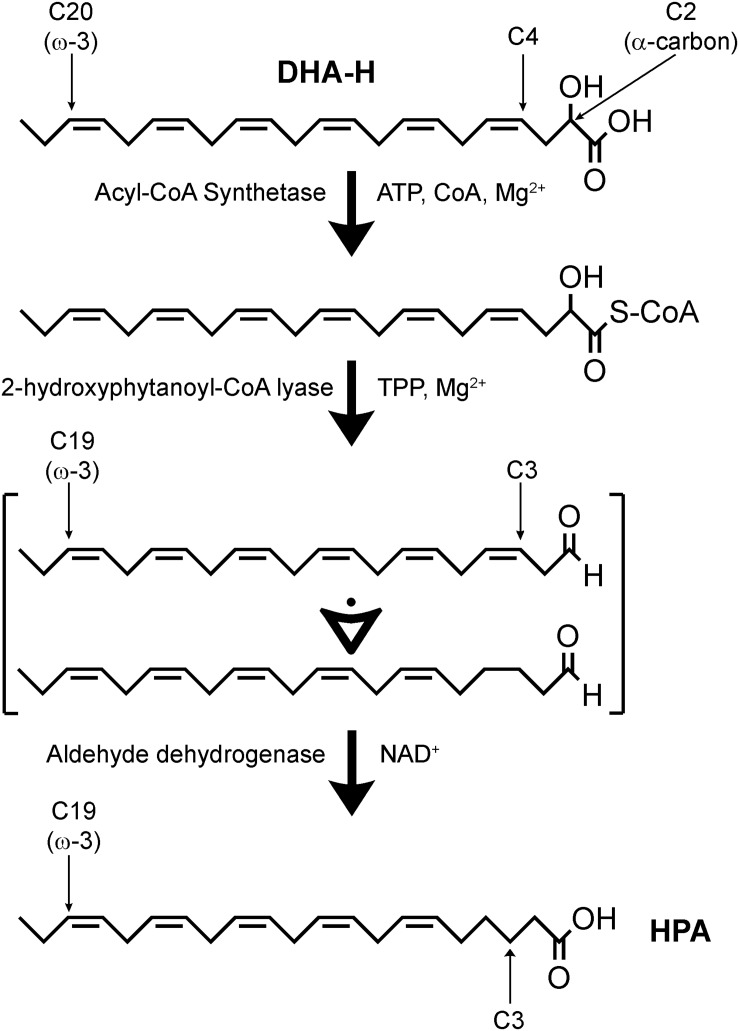
Hypothetic α-oxidation pathway for the conversion of DHA-H into HPA. DHA-H requires activation by an acyl-CoA synthetase. DHA-H-CoA would be subject to 2-hydroxyphytanoyl-CoA lyase (HACL1) activity, leading to the formation of an intermediate polyunsaturated fatty aldehyde which should contain 5 or 6 double bonds. It is not clear at what time the double bond originally located at DHA-H carbon 4 is lost. This process is dependent on thiamine pyrophosphate (TPP) and Mg^2+^, and it can be inhibited by the competitive antagonist, oxythiamine. Aldehyde dehydrogenase would be responsible for conversion of the intermediate aldehyde into HPA and the subsequent metabolism of HPA would take place via β-oxidation.

In a hypothetic pathway for the α-oxidation of DHA-H to produce HPA (Figure 2), DHA-H should be linked to CoA before HACL1 converts the 2-hydroxy fatty acid to an intermediate aldehyde containing an odd number of carbon atoms (Foulon et al., 2005). Aldehyde dehydrogenase would then modify this intermediate aldehyde into HPA, the corresponding fatty acid containing n-1 carbon atoms relative to DHA-H. In this context, it was notable that the undefined peak evident after DHA-H treatment (Figure 1A, panel a2, see asterisk) was also downregulated when oxythiamine was added to the cell culture medium along with DHA-H (compare panels a2 and a3). Hence, this molecule must also be produced by (or downstream of) HACL1 activity. The retention time of this molecule did not appear to correspond to any known fatty acid (as FAME) and thus, it must be a volatile molecule so as to be detected by GC. Accordingly, it was speculated that this molecule might be the intermediate aldehyde formed by HACL1 activity and reduced by aldehyde dehydrogenase to generate HPA (Figure 2). Otherwise, it remains unknown the exact molecular mechanism behind the reduction of the double bond originally located at the DHA-H carbon 4 (see Figure 2). We know that reduction happens when the double bond is located between carbons 4 and 5 but the double bond remains unaltered in 2-hydroxyoleic acid, which contains its unsaturation between carbons 9 and 10 (Guardiola-Serrano et al., 2019). Moreover, in DHA-H, the double bond between carbons 7 and 8 and further double bonds remain unaltered. These facts suggest that the proximity to the carboxylic and hydroxyl moieties is involved in desaturation of the double bond between C4 and C5 but it is unlikely that further double bonds could be reduced. Consequently, we speculate about a possible gradient for double bond reduction, where the probability for double bond reduction is higher when it is located close to the carboxyl group.

Finally, an extensive lipidomic study was performed in HEK293T samples under the same treatment conditions previously shown in Figure 1C (panels c1 and c2). DHA-H-treated cells for 24 h (30 and 100 μM) as well as their controls were subjected to methanolic lipid extraction before MS analysis. ESI-MS results were expressed as relative concentrations (mol% of membrane lipids), and the linked and free forms of DHA-H and HPA were considered as four separate lipid classes (Table 1). This allowed the direct assessment of incorporation of DHA-H and HPA into membrane lipids. Indeed, our data revealed incorporation of DHA-H and HPA into complex lipids occurred in a dose-dependent manner. After stimulation with 100 μM DHA-H, the relative level of HPA-containing lipids piled up to ca. 7 mol% of total membrane lipids. HPA incorporated practically into all lipid classes, mostly into PC (see Supplementary Table S1). In agreement with the GC-FID results (see Figure 1C), the levels of lipid species containing DHA-H were notably lower (1.1 mol% of membrane lipids upon 100 μM DHA-H stimulation) than those containing HPA (see Table 1 and Supplementary Tables S1, S2). In contrast, the fraction of DHA-H remaining as a free fatty acid was strongly higher as compared with HPA. For DHA-H, the ratio of free vs. linked forms accounted for 10.7 and 23.5% after 30 and 100 μM DHA-H treatment, respectively, while for HPA, the corresponding values remained below 0.1% (0.03 and 0.08% after 30 and 100 μM DHA-H treatment, respectively; Table 1). Therefore, we concluded that DHA-H confers resistance to incorporation into complex lipids via carbon 1 as compared with HPA, a non-hydroxylated long-chain PUFA. This effect may be due to the steric hindrance being exerted by the hydroxyl group located at the α-carbon, which is adjacent to the carboxylic group on carbon 1.

**TABLE 1:** ESI-MS characterization of lipid classes in DHA-H-treated HEK293T cells cultures.

	Control	DHA-H (30 μM, 24 h)	DHA-H (100 μM, 24 h)
Lipid class	Mean^a^ ± SEM^a^	Mean^a^ ± SEM^a^	Mean^a^ ± SEM^a^
LPC	0.474 ± 0.060	0.404 ± 0.003 NS	0.352 ± 0.012 NS
PC	48.154 ± 1.093	47.489 ± 1.595 NS	46.223 ± 1.181 NS
PC-O	9.542 ± 1.441	9.397 ± 0.973 NS	7.971 ± 0.972 NS
LPE	0.261 ± 0.032	0.208 ± 0.006 NS	0.212 ± 0.010 NS
PE	10.494 ± 1.077	10.267 ± 0.384 NS	11.220 ± 0.753 NS
PE-P	6.426 ± 0.434	4.829 ± 0.202*	4.157 ± 0.342**
LPI	0.127 ± 0.022	0.125 ± 0.021 NS	0.096 ± 0.012 NS
PI	7.871 ± 0.459	6.972 ± 0.366 NS	6.213 ± 0.248*
LPS	0.078 ± 0.009	0.061 ± 0.007 NS	0.053 ± 0.002 NS
PS	5.608 ± 0.314	5.076 ± 0.238 NS	4.875 ± 0.232 NS
LPG	0.006 ± 0.000	0.004 ± 0.000**	0.003 ± 0.000***
PG	0.402 ± 0.023	0.380 ± 0.020 NS	0.421 ± 0.021 NS
LPA	0.007 ± 0.001	0.005 ± 0.001 NS	0.003 ± 0.000*
PA	0.665 ± 0.042	0.521 ± 0.043*	0.427 ± 0.007**
CL	1.676 ± 0.114	1.617 ± 0.084 NS	1.494 ± 0.037 NS
SM	3.953 ± 0.126	4.100 ± 0.180 NS	4.163 ± 0.100 NS
Cer	0.278 ± 0.024	0.248 ± 0.012 NS	0.248 ± 0.025 NS
HexCer	0.497 ± 0.025	0.450 ± 0.021 NS	0.415 ± 0.015*
GM3	0.633 ± 0.065	0.649 ± 0.034 NS	0.644 ± 0.040 NS
DG	2.599 ± 0.217	2.764 ± 0.030 NS	2.656 ± 0.208 NS
TG	0.484 ± 0.012	0.633 ± 0.043*	0.687 ± 0.042**
CE	0.128 ± 0.025	0.132 ± 0.027 NS	0.103 ± 0.022 NS
DHA-H (linked)^b^	0.000 ± 0.000	0.438 ± 0.018 ***	1.121 ± 0.036 ***
HPA (linked)^b^	0.249 ± 0.049	3.996 ± 0.231 ***	7.032 ± 0.711 ***
DHA-H (free)^c^	0.000 ± 0.000	0.047 ± 0.007 NS	0.268 ± 0.056 ***
HPA (free)^c^	0.000 ± 0.000	0.001 ± 0.000 NS	0.005 ± 0.001 **
% of free/linked (DHA-H)		10.681 ± 1.380	23.511 ± 4.378
% of free/linked (HPA)		0.026 ± 0.009	0.080 ± 0.025

^*a*^Lipid classes are expressed as mol% of total membrane lipids (mean ± SEM). All data was normalized accordingly with the protein content in samples. ^*b*^DHA-H and HPA (linked) make reference to lipids containing DHA-H or HPA in their structures. ^*c*^DHA-H and HPA (free) make reference to the free fatty acid forms. ANOVA and Tukey’s post hoc test: NS, Not Significant. * p < 0.05, ** p < 0.01, and *** p < 0.001 as compared with control condition.

### DHA-H and DHA Are Incorporated and Accumulated Distinctly in HEK293T Cells

**FIGURE 3:**
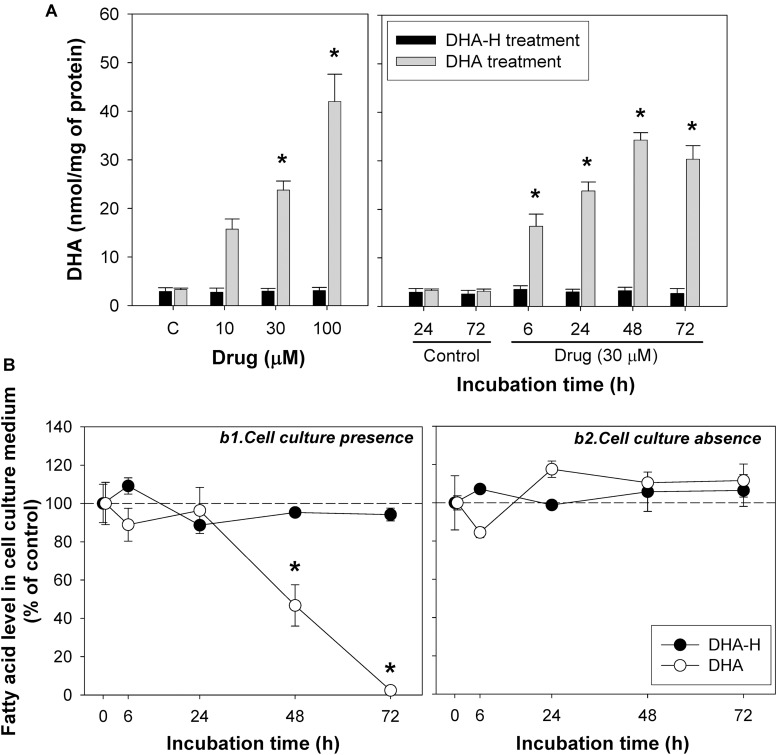
The incorporation and intracellular accumulation of DHA-H and DHA differs in HEK293T cells. **(A)** Intracellular DHA levels after treatment with DHA-H and the native form of DHA. DHA-H treatment had no effect on DHA levels whereas the native form of DHA significantly increases the DHA levels in a concentration- and time-dependent manner. **(B)** Levels of DHA-H and DHA in the cell culture medium in the presence (b1) or absence (b2) of cultured cells. The drug concentration in cell culture medium was 30 μM and the plates were incubated for up to 72 h. In the presence of the cells, the levels of DHA in the medium decreased significantly at 48 and 72 h as a result of DHA uptake by the cells, whereas the levels of DHA-H remained unaltered for up to 72 h. In the absence of cells, the levels of both drugs remained constant. The bars and points represent mean ± SEM, and the statistical analysis was performed by one-way ANOVA and with Tukey’s *post hoc* test: ^∗^*p* < 0.05 compared to the controls.

Exposure to increasing concentrations of DHA-H for different incubation times did not modify the intracellular levels of the natural analog DHA (docosahexaenoic acid; C22:6 n-3) in HEK293T cells, whereas exposure to non-hydroxylated DHA did induce a significant concentration- and time-dependent increase in intracellular DHA (Figure 3A) until a plateau was reached after 48 h. Similar results were obtained with N2a cells (Supplementary Figure S2). As expected, exposure to the non-hydroxylated form of DHA did not significantly modify the HPA levels, since this fatty acid is not metabolized by α-oxidation (Table 2). The preferential metabolic route for DHA must be β-oxidation, although other metabolic transformations cannot be ruled out. In fact, DHA can be converted into EPA (eicosapentaenoic acid, C20:5 n-3) (Park et al., 2016) and indeed, we detected a significant increase in EPA after DHA stimulation (see Table 2). Likewise, there was a similar upregulation of docosapentaenoic acid (DPA, C22:5 n-3) following DHA stimulation (see Table 2), suggesting that DHA could be converted into DPA. We also observed a significant downregulation of oleic acid (C18:1 n-9) following the exposure of the cells to DHA. Interestingly, none of these fatty acid modifications were observed after exposure to DHA-H (Table 2) and hence, the cellular fatty acid profile is differentially regulated by exposure to DHA-H and DHA. These differences may be explained, at least in part, by the different metabolic pathway each compound follows. Moreover, the different fatty acid profiles found following exposure to DHA-H and DHA may suggest these molecules drive different neuroprotective mechanisms. Although both DHA-H and DHA have demonstrated neuroprotective properties and both are structurally similar (Dyall, 2015; Torres et al., 2016), their distinct metabolism generates different metabolites and produces different changes in membrane lipid composition, properties that in turn suggest the triggering of different molecular mechanisms in both cases.

**TABLE 2:** GC-FID characterization of fatty acid profile in DHA-H- and DHA-treated HEK293T cell cultures.

	Control	DHA-H (30 μM, 24 h)	DHA-H (100 μM, 24 h)	DHA (30 μM, 24 h)	DHA (100 μM, 24 h)
Fatty acid	Mean^a^ ± SEM^a^	Mean^a^ ± SEM^a^	Mean^a^ ± SEM^a^	Mean^a^ ± SEM^a^	Mean^a^ ± SEM^a^
2-OH 22:6 n-3	0.00 ± 0.00	0.21 ± 0.06**	0.74 ± 0.09***	0.00 ± 0.00 NS	0.00 ± 0.00 NS
22:6 n-3	3.10 ± 0.21	2.99 ± 0.23 NS	3.11 ± 0.21 NS	23.80 ± 1.85***	42.01 ± 5.62***
21:5 n-3	0.09 ± 0.01	1.74 ± 0.25***	4.01 ± 0.43***	0.20 ± 0.04 NS	0.52 ± 0.11 NS
22:5 n-3	1.94 ± 0.14	1.78 ± 0.20 NS	1.91 ± 0.22 NS	3.21 ± 0.35**	3.50 ± 0.35***
20:5 n-3	2.01 ± 0.10	1.80 ± 0.13 NS	1.97 ± 0.13 NS	6.99 ± 0.49***	6.16 ± 0.96***
20:4 n-6	5.74 ± 0.29	5.32 ± 0.50 NS	5.54 ± 0.53 NS	5.53 ± 0.84 NS	5.06 ± 0.84 NS
18:2 n-6	1.48 ± 0.16	1.40 ± 0.20 NS	1.50 ± 0.22 NS	1.31 ± 0.04 NS	1.40 ± 0.14 NS
18:1 n-9	39.74 ± 1.89	35.84 ± 2.75 NS	39.72 ± 3.41 NS	22.71 ± 2.74**	18.20 ± 2.52***
18:0	32.19 ± 4.70	30.07 ± 1.70 NS	33.51 ± 1.27 NS	33.61 ± 0.50 NS	33.34 ± 0.52 NS
16:0	42.27 ± 2.36	40.26 ± 3.88 NS	45.36 ± 4.62 NS	46.67 ± 3.18 NS	46.60 ± 4.41 NS

^*a*^Fatty acid levels (mean ± SEM) are shown as nmol/mg of protein. Multiple statistical analysis was performed with one-way ANOVA & Tukey’s post hoc test: NS, Not Significant. * p < 0.05, ** p < 0.01, and *** p < 0.001 as compared with control condition.

It is noteworthy that under the same conditions, exposure to DHA increased DHA levels more than exposure to DHA-H increased the levels of DHA-H and HPA (see Figure 1A, compare panels a2 and a4): exposure to DHA (100 μM for 24 h) increased the amount of DHA from 3.10 ± 0.21 to 42.01 ± 5.62 nmol/mg of protein, while DHA-H treatment (100 μM for 24 h) increased the DHA-H levels from 0 to 0.74 ± 0.09, and those of HPA from 0.09 ± 0.01 to 4.01 ± 0.43 nmol/mg of protein. Although the basal levels of DHA and HPA (see Table 2) were subtracted from the levels quantified after DHA and DHA-H stimulation, respectively, the amount of DHA detected was still almost one order of magnitude higher than the levels of HPA and DHA-H together. This fact may be due to the more rapid catabolism of DHA-H and its metabolite HPA, or to the more rapid DHA uptake than that of DHA-H. These possibilities are not mutually exclusive, although it is notable that DHA and HPA are both fatty acids that are preferentially catabolized by β-oxidation, so they are likely to oxidized to a similar extent under the same experimental conditions. In addition, the culture medium for 293T cells is supplemented with high concentrations of glucose, which would not favor β-oxidation of fatty acids by these cells (see section Materials and Methods) (Reddy and Hashimoto, 2001). Thus, it seems more likely that the aforementioned differences reflect distinct rates of incorporation into the cells. To test this hypothesis, we assessed the levels of DHA and DHA-H in the cell culture medium over time, in the presence or absence of cultured 293T cells. Aliquots of the medium were removed at 6, 24, 48, and 72 h, and with cell confluence not reached at any of these time points (Figure 3B). The results demonstrated that DHA is more strongly taken up by the cells than DHA-H. The DHA levels remained quite constant in the culture medium until 24 h but then decreased to 46.74 ± 10.76% at 48 h, and to 2.40 ± 0.14% at 72 h (relative to the initial amount added to the cultures considered as 100%, time 0). By contrast, the DHA-H levels remained practically unaltered up to 72 h. Moreover, in the absence of the cultured cells, the levels of both drugs in the medium remained constant over time, demonstrating that both lipids are stable at 37°C for 72 h. These results may explain the saturation effect previously observed in intracellular DHA levels after DHA stimulation between 48 and 72 h of incubation (see Figure 3A and Supplementary Figure S2). Therefore, these results suggest a preference of 293T cells for DHA with respect to DHA-H, which in turn should at least partially explain the differences between the DHA and DHA-H plus HPA quantified after exposure to DHA and DHA-H, respectively.

### Administration of DHA-H Leads to Elevated Levels of HPA in Blood Plasma and Brain

**FIGURE 4:**
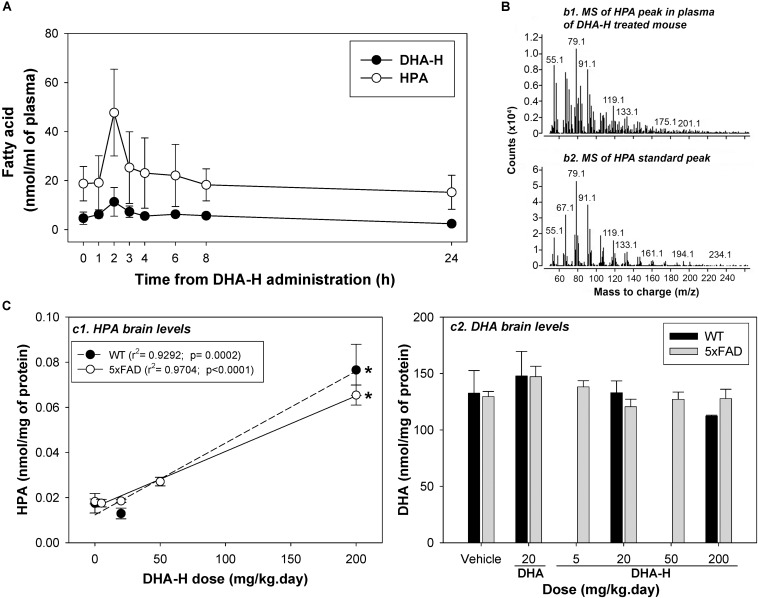
DHA-H administration leads to elevated HPA levels in mouse blood plasma and brain. **(A)** Steady state levels of DHA-H and HPA were determined in the blood plasma in WT mice administered DHA-H (15 daily doses at 200 mg/kg). Both molecules had a similar PK profile, with the notably higher levels of HPA than DHA-H. The maximum plasma concentration was reached 2 h after oral administration of the last dose. **(B)** Mass spectra for the HPA in mouse plasma (2 h after the 15th dose of DHA-H) and for the HPA standard. The main fragments in both spectra had the same mass-to-charge ratio (m/z). **(C)** The levels of HPA and DHA were determined in the brain of WT and 5xFAD mice after chronic administration of DHA-H. HPA accumulated in brain of both mouse strains in accordance with the DHA-H dose administered (c1), whereas DHA levels did not vary significantly among the experimental conditions (c2). A modest increase in DHA relative to the controls (vehicle) was only evident in mice administered DHA (20 mg/kg). The data are shown as the mean ± SEM, and the statistical analysis was performed by one-way ANOVA and with Tukey’s *post hoc* test: ^∗^*p* < 0.05 compared to control (vehicle-treated mice).

To investigate the role played by HPA in the therapeutic effect of DHA-H *in vivo*, we assessed the metabolism of DHA-H and the production of HPA in WT and 5xFAD mice. The latter an AD mouse model in which DHA-H has widely demonstrated neuroprotective effects (Fiol-Deroque et al., 2013; Torres et al., 2014; Mohaibes et al., 2017). Initially, the levels of DHA-H and HPA were assessed in the blood plasma of WT mice administered DHA-H over 15 days with a repeated dose of 200 mg/kg daily. As chronic administration with repeated daily doses was studied, the drug levels in plasma should correspond to steady-state levels and the drug intake should be in dynamic equilibrium with its elimination. After 14 days of drug administration, several blood samples were collected before/after the 15th dose at different times in order to characterize the steady state PK profile of DHA-H (Figure 4A). Both DHA-H and HPA had similar PK profiles, although HPA levels were considerably higher than those of DHA-H, consistent with the data from HEK293T cells (Figure 1). Hence, the conversion of DHA-H to HPA is an early event *in vivo*, probably mediated at the enteric and hepatic levels before reaching the brain. The highest steady state concentrations of both DHA-H and HPA (Cmax, ss) were reached 2 h after oral administration of DHA-H, with around fourfold more HPA than DHA-H. DHA-H was readily detected 2 h after administration but it was not easy to detect at other times, even though the dose of DHA-H administered was 10-fold higher than the therapeutic dose previously described (20 mg/kg.day) (Fiol-Deroque et al., 2013; Torres et al., 2014) and the animals had been treated for14 days before tracing the PK profile. The chromatographic peak that corresponded to HPA in plasma 2 h after DHA-H administration was subjected to GC-MS to confirm the molecular identity of this DHA-H metabolite *in vivo* (Figure 4B). As expected, the mass spectrum of the HPA peak in plasma displayed the main ionic fragments of the standard HPA mass spectrum, demonstrating that HPA was also produced in mice like in cultured cells (see Figure 1B). Finally, after 2 h there was a consistent loss of HPA and 24 h after DHA-H administration, the steady state levels of HPA were similar to those observed just before DHA-H administration (time 0).

We also measured the brain levels of HPA (target organ) in WT and 5xFAD mice after 4-months of chronic daily DHA-H administration (Figure 4C). Notably, DHA-H remained undetectable and HPA was only detected clearly at the highest doses of DHA-H administered (50 and 200 mg/kg). The chromatographic peak identified as HPA in the brain was evident at basal levels (without DHA-H treatment), although it was not confirmed that this peak truly belonged to HPA (see discussion below). In any case, there was a significant linear correlation of the brain HPA levels to DHA-H administered to both the WT and 5xFAD mice (Figure 4C, panel c1), and no remarkable differences were found between WT and 5xFAD in these assays. In fact, following the same conditions of DHA-H administration, similar levels of HPA were found in both strains. Furthermore, to check if the neuroprotective effect of DHA-H is mediated by an increase in DHA levels, brain levels of DHA were also measured in WT and 5xFAD following DHA-H and DHA administration (Figure 4C, panel c2). Interestingly, the DHA levels recorded were similar in all the different experimental conditions. Thus, treatment of WT and 5xFAD mice with DHA-H did not significantly modify the levels of DHA relative to the controls (vehicle). Moreover, the treatment with a therapeutic dose of DHA (20 mg/kg) induced only a modest but not significant increase in the average levels of DHA in both WT and 5xFAD mice. Unlike HEK293T cells, the levels of DHA were notably high in the brain (around 5 nmol/mg of protein in cells as opposed to more than 100 nmol/mg of protein in the brain) and thus, the effect of DHA stimulation on DHA brain levels might be masked by the naturally high levels of DHA present in the brain. In summary, administration of DHA-H to mice induced a significant increase in brain HPA levels, whereas DHA-H remained undetectable and the DHA levels unmodified. These data point to HPA as an effector of the therapeutic effects of DHA-H against the AD neuropathology in 5xFAD mice.

### Brain HPA Levels Are Directly Correlated With Cognitive Spatial Memory Abilities in 5xFAD Mice

**FIGURE 5:**
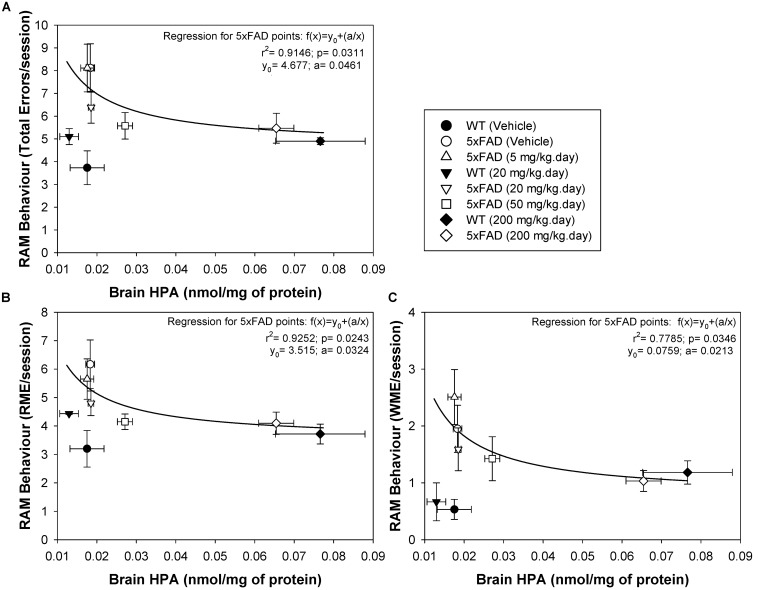
Brain HPA levels in 5xFAD mice are positively correlated with spatial memory performance. The number of total errors **(A)**, RME **(B)**, and WME **(C)** committed were plotted against the HPA brain levels. Individual experimental points from the complete animal population studied were plotted and adjusted to an inverse polynomial regression. The r^2^ and p regression values are shown for each RAM parameter: total errors, RME and WME, against HPA brain concentration. The data obtained suggest that minimal increases in HPA brain levels are associated with an improvement in spatial cognition. Each point represents the mean ± SEM for each treatment.

The radial arm maze (RAM) is a spatial learning and memory test that allows both working and reference memory to be assessed (Wirsching et al., 1984), reflecting both hippocampal and frontal activity (Hasselmo and Howard, 2005; Martin and Clark, 2007). This test was previously used to assess the effect of DHA-H on 5xFAD spatial behavior, demonstrating a clear therapeutic effect of DHA-H at a daily dose ranging from 20 to 200 mg/kg with no apparent toxic effects (Torres et al., 2015; Mohaibes et al., 2017). Here, we correlated the brain levels of HPA in WT and 5xFAD mice with their performance in the RAM, assessing the total errors, the RMEs and the WMEs (Figures 5A–C). To demonstrate a direct relationship between spatial cognitive scores (an inverse relationship between the number of errors committed) and brain HPA levels in 5xFAD mice, all individual experimental points were adjusted to an inverse polynomial first order regression instead of a linear regression. The relationship between the RAM parameters measured and the brain HPA levels best adjusted to the polynomial inverse regression, because the therapeutic dose of 20 mg/kg DHA-H improved spatial behavior, while the HPA levels at this dose were virtually identical to the basal levels in vehicle-treated 5xFAD mice or those that received 5 mg/kg. Similarly, a dose of 50 mg/kg dose also affected the number of errors produced, while HPA levels only increased minimally from the basal levels. Under these circumstances, the experimental points fitted the polynomial inverse regression but not the linear regression (Figure 5). Moreover, the significant adjustment of the experimental points to this type of regression suggested that spatial cognition improved exponentially with the increase in HPA brain levels, such that minimal increases in HPA levels in the brain would provoke neuroprotective effects. In this context, the total errors and RMEs were the RAM parameters that best fitted the inverse regression for 5xFAD mice whereas WMEs displayed a lower adjusted r^2^ parameter relative to the total errors or RMEs (see Figure 5C). In the case of WT mice, none of the experimental points adjusted to any regression. All of them, vehicle, 20 and 200 mg/kg treated mice committed a similar number of errors, such that the number of errors committed by WT mice established the maximum level of spatial cognitive recovery in 5xFAD mice administered DHA-H. Indeed, it is noteworthy that the number of errors committed by 5xFAD mice administered 20–200 mg/kg DHA-H were quite similar to those of WT mice, particularly in terms of total errors and RME, demonstrating the therapeutic effect of DHA-H on the spatial performance of 5xFAD mice. In summary, the data presented here indicated that HPA brain levels were closely related to the spatial cognitive improvement in 5xFAD mice, indicating that HPA could drive therapeutic effect of DHA-H in AD, and that HPA may be a biomarker to monitor DHA-H therapy in humans.

## Discussion

Alzheimer’s disease is a neurodegenerative condition for which there is currently no plausible treatment. The only drugs available for AD therapy do not prevent neurodegeneration and they were approved more than one decade ago. Despite all the efforts in the field and the wide collection of new drugs and therapies developed (including immunotherapies), none of these have passed phase-III clinical trials. As a result, it has been questioned if the amyloid cascade hypothesis should still be used to design new drugs and therapies, and more and more researchers have started assessing new therapeutic approaches based on alternative perspectives on the AD pathophysiology. Here, a membrane lipid therapy perspective has been adopted to assess an AD therapy, assuming that the main events triggering the pathophysiology of AD are related to alterations in membrane lipid composition and structure (Escribá et al., 2008, 2015). DHA-H, a hydroxylated derivative of DHA, is a drug under pre-clinical development which have widely demonstrated potential therapeutic effects in AD and PD (Fiol-Deroque et al., 2013; Torres et al., 2014, 2015; Mohaibes et al., 2017; Hernando et al., 2019). Here, we demonstrate that: (i) DHA-H is metabolically converted into HPA via α-oxidation; (ii) hydroxylation of DHA at the α-carbon confers resistance to its incorporation into other lipids and promotes its accumulation as a free fatty acid in cell membranes; (iii) DHA-H and DHA accumulate distinctly in cells and they regulate cellular lipid composition; (iv) hydroxylation of DHA at the α-carbon hinders its drug incorporation into cultured cells; (v) mouse administration of DHA-H leads to an increase in plasma HPA levels over and above those of DHA-H; (vi) brain DHA levels are not significantly modified by DHA-H administration; (vii) only HPA, and not DHA-H, is detected in the DHA-H-treated mouse brain; and (viii) HPA brain levels are closely related to improved spatial memory in 5xFAD mice.

HPA was first observed in cultured cells treated with DHA-H and the presence of this metabolite in DHA-H-treated cells was seen here in both HEK293T and mouse N2a neuroblastoma cells. The detection of HPA in two such distinct cell types suggests that DHA-H metabolism to HPA is likely to be a constitutive pathway present in most cell types. Here, we demonstrate that this pathway involves α-oxidation mediated by HACL1, an enzyme found in peroxisomes, and dependent on thiamine pyrophosphate and Mg^2+^ (Foulon et al., 1999). In fact, this route has been implicated previously in α-oxidation of short/medium 2-hydroxy straight-chain fatty acids and 2-hydroxyoleic acid (Foulon et al., 2005; Guardiola-Serrano et al., 2019), yet until now in long-chain PUFAs like DHA-H. In accordance with the results reported here, α-oxidation appears to be the main pathway involved in DHA-H metabolism leading to HPA, via an intermediated fatty aldehyde (Foulon et al., 2005; Jansen and Wanders, 2006). HPA produced by DHA-H α-oxidation can then follow 2 pathways: cell accumulation as part of complex lipids like phospho- or glycerolipids (Larsen et al., 1997), or catabolism via β-oxidation. Our results demonstrate that at least part of the HPA produced accumulates in a concentration- and time-dependent manner (see Figure 1C), mostly as PC species (see Supplementary Table S1). It should be noted that these assays have been developed in culture medium supplemented high glucose concentrations (for HEK293T cells) or insulin (for N2a cells). Under these conditions, fatty acid β-oxidation is unlikely to be favored (Hamel et al., 2001; Reddy and Hashimoto, 2001), such that the HPA detected in such experiments may mainly be produced by DHA-H α-oxidation with only a minimal influence because of HPA catabolism. Finally, it is of note that absolute levels of DHA-H and HPA were quantified according to a C17:0 free fatty acid standard (see Figure 1C), although taking into consideration our lipidomic analysis (see Supplementary Tables S1, S2), a better standard for this purpose would be a diacyl-17:0-PC instead of the free fatty acid.

Interestingly, intracellular DHA levels were almost one-magnitude-order higher than the levels of DHA-H and HPA together, following exposure to DHA and DHA-H, respectively. This difference could be explained because of distinct DHA-H and DHA metabolism. DHA-H is α-oxidized to HPA which in turn is β-oxidized up to propionyl-CoA, which in turn is converted into succinyl-CoA (process dependent on biotin and vitamin B12) which enters the tricarboxylic acid (TCA or Krebs) cycle (Bhagavan and Ha, 2011; Jenkins et al., 2015). Alternatively, DHA is simply β-oxidized to acetyl-CoA, which enters the TCA cycle (Reddy and Hashimoto, 2001). However, given that both HPA and DHA should be degraded equally by β-oxidation, and that β-oxidation should not be favored under the culture conditions used, metabolic differences are unlikely to explain this effect. The distinct molecular structure and polarity of these two molecules may also explain the differences in their accumulation, and hydroxylation of DHA could interfere with its uptake by cells in culture. Indeed, DHA-H is not incorporated into the cells as efficiently as the natural analog DHA, which probably explains the distinct intracellular levels of DHA and, DHA-H plus HPA, after exposure to DHA and DHA-H, respectively. Since the mechanisms involved in fatty acid uptake are not fully understood, it may be difficult to elucidate the reasons underlying the preferential uptake of DHA by cells in culture. Regarding fatty acid incorporation by passive diffusion across the cell membrane, the increased polarity of DHA-H due to its hydroxylation could interfere with its passive diffusion (Hamilton et al., 2001). Furthermore, many proteins are involved in active fatty acid transport across the plasma membrane, such as the acyl-CoA synthetases implicated in long-chain fatty acid (LCFA) uptake (Mashek et al., 2007). In humans, two acyl-CoA synthetase families activate long chain fatty acids: the fatty acid transport proteins (FATPs) and the long chain acyl-CoA synthetases (ACSLs). These families differ in their tissue expression and cellular location, and they channel LCFAs into the cells together with the scavenger CD36 (Mashek et al., 2007; Krammer et al., 2011; Schneider et al., 2014). In this sense, if DHA-H uptake is slower than DHA uptake, it may be at least in part due to the α-hydroxyl group of the DHA-H molecule provoking steric hindrance to acyl-CoA synthetases. These enzymes catalyze the thio-esterification of the fatty carboxyl group to CoA, and the hydroxyl group lies just adjacent to the carboxyl group in DHA-H, such that the proximity of both chemical groups might account for the poor esterification of CoA and hence, its lower uptake. This hypothesis is supported by the results obtained from the lipidomic analysis carried out in DHA-H treated cells (see Table 1). These results demonstrated that hydroxylation at the α-carbon leads to steric hindrance to carbon-1 linkage to other complex lipids. Therefore, this steric hindrance on carbon 1 may be extensive to the linkage with other molecules such as CoA.

The characterization of DHA-H and HPA levels in the treated mice confirmed the data obtained from cells in culture. Evaluating the levels of DHA-H and HPA in blood plasma, both molecules have a similar steady state PK profile with notably higher levels of HPA than DHA-H (Figure 4A). These results suggest that the conversion of DHA-H into HPA in animals must be an early event at the gastrointestinal tract and liver after oral administration. In fact, resistance to esterification displayed by DHA-H may explain why HPA levels were consistently higher than those of DHA-H in blood plasma. Absorbed fatty acids at gastrointestinal tract must be esterified into triglycerides (TGs), which can later be packaged into chylomicrons. The steric hindrance exerted by α-hydroxyl group on carbon 1 may delay incorporation of DHA-H into TGs, favoring its α-oxidation in enteric cells to produce HPA, which in turn should be preferentially incorporated into TGs and packaged into chylomicrons. It must not be ruled out that one fraction of DHA-H could be transported bound to albumin and other fraction must be esterified to form TGs and transported by lipoproteins. Resistance of DHA-H to be incorporated into TGs could favor its transport as a free acid bound to albumin. This portion of DHA-H is also susceptible to be metabolically converted into HPA by the liver. Finally, the fraction of DHA-H which is esterified into TGs may be partially protected from α-oxidation because DHA-H carbon 1 is esterified. However, taking into account the presence of hepatic lipoprotein lipases, at least one part of this esterified DHA-H is likely released from TGs, leaving carbon 1 free and allowing DHA-H α-oxidation, in the liver. Therefore, although the proportions of DHA-H being converted into HPA, transported with albumin or packaged into chylomicrons are unknown, the molecular structure of DHA-H and, particularly, the steric hindrance exerted by the hydroxyl group adjacent to carbon 1 would be able to explain the early conversion of most DHA-H into HPA shown in blood plasma.

In addition, while there was a dose-dependent accumulation of HPA in the brain of mice chronically administered DHA-H, DHA-H remained undetectable even when administered at high doses. Similar results were obtained in both WT and 5xFAD mice, which in turn suggest that BBB permeability is not altered in 5xFAD mice at time of sacrifice (see Materials and Methods). Since DHA-H was not detected in the brain, it remains unclear whether DHA-H is not present in that organ or if it is present below threshold levels. In this respect, the metabolism of DHA-H and the specificity of BBB transporters like Mfsd2a may account for the difficulties in detecting DHA-H in the brain (Nguyen et al., 2014; Almutairi et al., 2016). Conversely, HPA could be measured in the mouse brain following DHA-H administration, and basal levels could even be detected in mice administered the vehicle alone. We did not confirm here that the basal HPA detected in the brain (or culture cells) corresponded to the molecular structure of HPA because basal levels of HPA in humans have been reported previously (Masquio et al., 2016). However, our preliminary GC-MS study failed to detect HPA in the human brain (unpublished results), which in turn raises questions as to whether HPA is present physiologically in the brain. Therefore, further research into this issue is necessary to determine whether this C21:5 n-3 fatty acid is actually present in the human brain and how its levels are modified as AD progresses, particularly since omega-3 PUFAs like DHA and EPA are downregulated in AD, and they have been proposed as AD biomarkers (Chan et al., 2012; Dyall, 2015). Regardless of the value of HPA as a biomarker in AD, the data presented here clearly demonstrate the close relationship between DHA-H treatment and the HPA in blood and brain, so that HPA is likely to be a suitable biomarker for future DHA-H therapy in humans.

The implications of DHA-H metabolism in the central nervous system (CNS) has been assessed in relation to neuroprotection and its possible toxic effects. It has been assumed that DHA-H would enrich brain membranes in PE species carrying DHA, which in turn would promote liquid-disordered prone structures with beneficial effects to combat AD (Vetrivel and Thinakaran, 2010; Shaikh, 2012). This hypothesis was based on a lipidomic analysis of the 5xFAD mouse brain following DHA-H administration (Torres et al., 2014). However, here it appears that DHA levels are not significantly modified in the brain of WT or 5xFAD mice following DHA-H administration under similar conditions (Fiol-Deroque et al., 2013; Torres et al., 2014; Mohaibes et al., 2017). Therefore, the enrichment of brain membranes in highly polyunsaturated PE species must be mediated by an increase in PUFAs other than DHA and probably those derived from the HPA. Indeed, we see a significant positive correlation between brain HPA levels and spatial memory in AD mice, suggesting that HPA is a DHA-H effector in the CNS, particularly as DHA-H could not be detected and DHA levels are not significantly modified in the 5xFAD mouse brain. Hence, further studies will be necessary to address the effect of HPA on β-amyloid accumulation and tau phosphorylation in order to stablish if DHA-H conversion into HPA is responsible for the therapeutic effect of DHA-H. In this context, the evidence that DHA-H is metabolized distinctly to DHA may reflect different means of neuroprotection. We have demonstrated here that exposure to DHA mainly enriches cells in DHA and EPA, whereas DHA-H has no such an effect. Both these omega-3 PUFAs are tightly regulated in the CNS (Chen et al., 2009; Chen and Bazinet, 2015) and it is likely that these mechanisms of control are affected by the AD pathophysiology. In this sense, DHA-H conversion into an odd-chain omega-3 PUFA like HPA could offer certain therapeutic benefits since HPA could escape from the mechanisms of DHA/EPA regulation, allowing the correct proportion of omega-3 PUFAs to be reestablished in the brain, which in turn should recover the omega-3 PUFA activity in the CNS. Alternatively, the metabolic differences between DHA-H and DHA may have an effect on possible toxic effects selectively associated to DHA-H and not DHA. As mentioned, HPA is β-oxidized up to propionyl-CoA whereas DHA is β-oxidized to acetyl-CoA. While propionyl-CoA must undergo enzymatic conversion to succinyl-CoA in order to enter the TCA cycle, the pathological accumulation of propionyl-CoA may produce propionic acidemia. This disorder can be corrected in part by vitamin B12 administration and dietary protein restriction (since propionate is primarily derived from amino acids) (Bhagavan and Ha, 2011; Jenkins et al., 2015). Consequently, propionic acidemia may appear following DHA-H administration but not that of DHA, and patients with congenital difficulties in converting propionyl-CoA to succinyl-CoA should avoid this compound.

## Conclusion

In conclusion, we address here certain metabolic issues associated with DHA-H treatment, highlighting the differences between administering the hydroxylated and non-hydroxylated forms of DHA. DHA hydroxylation on carbon 2 resulted in its enzymatic conversion to HPA via α-oxidation, a product that accumulates in cultured cells, in blood plasma and in the brain after exposure to DHA-H. Interestingly, HPA is an omega-3 PUFA with an odd number of carbons, which might induce neuroprotection through different pathways to those driven by DHA. This metabolic conversion could offer advantages in AD therapy relative to DHA and EPA administration, which have produced little clinical improvement in several clinical trials on human patients (Freund-Levi et al., 2006; Quinn et al., 2010). Indeed, HPA accumulation in the brain is correlated with the spatial performance of 5xFAD mice, apparently in the absence of DHA-H and with no significant changes in DHA levels. Therefore, the metabolic conversion of DHA-H into HPA could represent a key molecular event that in part explains the neuroprotective and neuroregenerative effects of DHA-H, and its therapeutic benefits in AD relative to other known therapies (including DHA).

## Data Availability Statement

The datasets generated for this study are available on request to the corresponding authors.

## Ethics Statement

The animal study was reviewed and approved by *Comité de Ética en Experimentación Animal de la Universidad de las Islas Baleares* (CEEA-UIB) & *Govern de les Illes Balears* (Regional Government of the Balearic Islands, Spain).

## Author Contributions

PE: drug design and synthesis. MT and PE: experimental design. SP, ÁI, LA, MM, and MT: data acquisition and analysis of HEK293T cells. SP, MO, and MT: data acquisition and analysis of N2a cells. SP, ÁI, MO, JC, PF-G, VL, and MT: data acquisition in the animal studies. MP, GB, and MT data acquisition and analysis in the lipidomic study. MT and PE: data interpretation. MT: writing of the manuscript. MT and PE: response to reviewers. MT, PE, and XB: revision of the manuscript.

## Conflict of Interest

MT, PF-G, VL, XB, and PE declare that they are shareholders in several biotech companies: Laminar Pharmaceuticals (MT, PF-G, VL, XB, and PE), Pharmaconcept (XB and PE), Neurofix (PE), and Ability Therapeutics (PE). VL was supported by a Torres-Quevedo Research Contract granted to Laminar Pharmaceuticals, S.L., from the *Ministerio de Economía y Competitividad* (Spanish Government). The remaining authors declare that the research was conducted in the absence of any commercial or financial relationships that could be construed as a potential conflict of interest.

## Footnotes

1 https://www.jax.org/news-and-insights/2005/october/how-much-blood-can-i-take-from-a-mouse-without-endangering-its-health

## Abbreviations

AD: Alzheimer’s disease
DHA-H: 2-hydroxy-docosahexaenoic acid
HPA: Heneicosapentaenoic acid
DHA: Docosahexaenoic acid
WT: Wild-type
RAM: Radial Arm Maze
RME: Reference Memory Errors
WME: Working Memory Errors
BBB: Blood Brain Barrier
CNS: Central Nervous System
EPA: Eicosapentaenoic acid
PUFA: Polyunsaturated Fatty Acids
FAME: Fatty Acid Methyl Ester
GC-FID: Gas Chromatography-Flame Ionization Detector
GC-MS: Gas Chromatography-Mass Spectrometry
ESI-MS: Electrospray ionization-Mass Spectrometry
HACL1: 2-hydroxyacyl-CoA lyase 1 or 2-hydroxyphytanoyl-CoA lyase
PK: Pharmacokinetic
CoA: Coenzyme A
PE: Phosphatidylethanolamine diacyl
PE-P: Phosphatidylethanolamine alkenyl-acyl (plasmalogen)
PC: Phosphatidylcholine diacyl
PC-O: Phosphatidylcholine alkyl-acyl
LPC: lysophosphatidylcholine
SM: sphingomyelin
DG: diacylglycerol
TG: triacylglcerol
CE: Cholesteryl ester
LPE: lysophosphatidylethanolamine
PI: phosphatidylinositol
LPI: lysophosphatidylinositol
PS: phosphatidylserine
LPS: lysophosphatidylserine
PA: phosphatidic acid
LPA: lysophosphatidic acid
PG: phosphatidylglycerol
LPG: lysophosphatidylglycerol
CL: cardiolipin
Cer: ceramide
HexCer: Hexosyl ceramide
GM3: ganglioside GM3.
