# Supplementary material for: 2-Hydroxy-Docosahexaenoic Acid Is Converted Into Heneicosapentaenoic Acid via α-Oxidation: Implications for Alzheimer’s Disease Therapy
Source: Front Cell Dev Biol. 2020 Mar 27;8:164. doi: 10.3389/fcell.2020.00164 (PMC7122748; doi:10.3389/fcell.2020.00164)
Supplement: Supplementary file 1 [file Data_Sheet_1.PDF]

Supplementary Material

Supplementary Figures

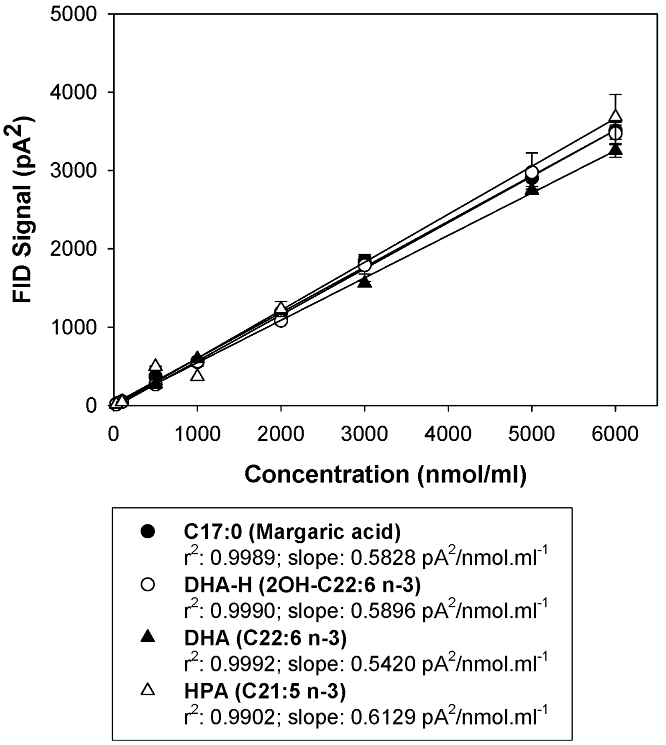

Supplementary Figure 1. Linear correlation between the peak area (FID Signal) and the concentration of fatty acid analyzed by GC-FID. Increasing concentrations of four different fatty acids: C17:0 (margaric acid), DHA-H (2-hydroxy-docosahexaenoic acid; 2OH-C22:6 n-3), DHA (docosahexaenoic acid; C22:6 n-3) and HPA (Heneicosapentaenoic acid; C21:5 n-3) were analyzed by GC-FID. All compounds were chemically derivatized to fatty acid methyl esters prior to analysis. The four fatty acids analyzed were very different in terms of structure (saturated and polyunsaturated acyl chains, with or without hydroxylation), yet they all displayed a very similar FID response along the same concentration gradient. C17:0 (n=6), DHA-H (n=4), DHA (n=2), HPA (n=2).

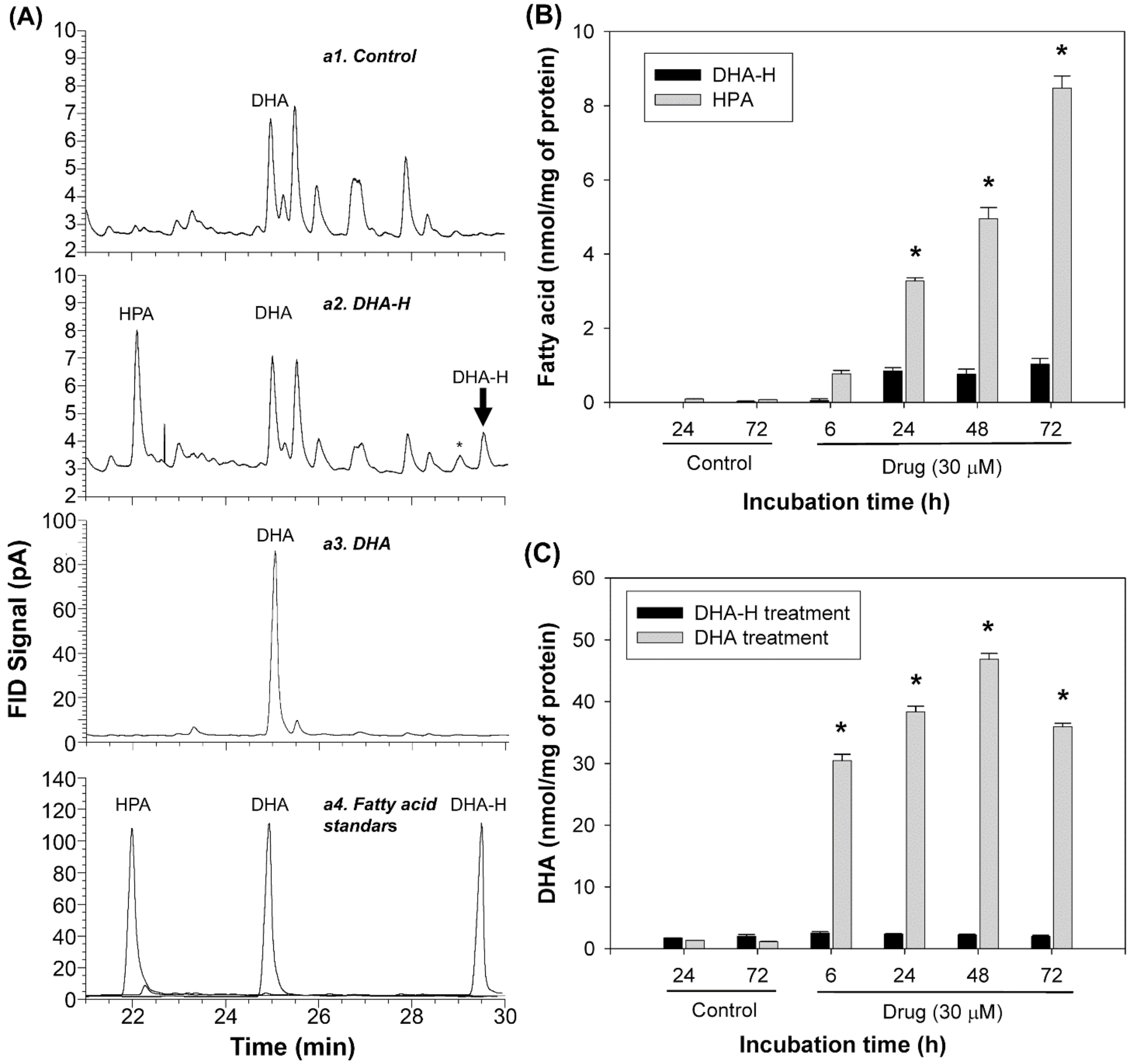

Supplementary Figure 2. DHA-H is converted into HPA in N2a cells. (A) Amplified regions of representative GC-FID chromatograms showing the fatty acid composition in different experimental conditions: control (a1), DHA-H treatment (100 µM, 24 h - a2), DHA (100 µM, 24 h - a3) and fatty acid standards (a4). Peaks of interest were identified based on their retention time and the peak marked with an asterisk is modulated by DHA-H but its identity is unknown. (B) Intracellular levels of DHA-H and HPA in cells exposed to DHA-H (30 µM) for different times. There is a time-dependent increase in DHA-H and HPA, yet with significantly higher levels of HPA than DHA-H from 24 hours onwards. (C) Exposure to DHA-H had no effect on the intracellular levels of DHA whereas exposure to the native DHA significantly increased the intracellular DHA in a time-dependent manner relative to the untreated controls. The bars represent the mean ± SEM, and the statistical analysis was performed with one-way ANOVA and with Tukey's post-hoc test: *, $p < 0.05$ when comparing HPA and DHA-H levels in panel (B); *, $p<0.05$ compared to control conditions in panel (C).

Supplementary tables

Supplementary table 1. ESI-MS characterization of HPA-containing lipid species in DHA-H-treated HEK293T cells cultures.

	Control			DHA-H (30 µM, 24h)				DHA-H (100 µM, 24h)			
**Lipid species[2]**	**Mean[1]**	**±**	**SEM[1]**	**Mean[1]**	**±**	**SEM[1]**		**Mean[1]**	**±**	**SEM[1]**	
LPC(21:5)	0.000	±	0.000	0.001	±	0.000	NS	0.002	±	0.001	*
PC(37:6)	0.007	±	0.002	0.188	±	0.046	**	0.334	±	0.021	***
PC(37:5)	0.012	±	0.004	0.547	±	0.028	***	1.369	±	0.106	***
PC(39:7)	0.006	±	0.002	0.035	±	0.005	***	0.056	±	0.001	***
PC(39:6)	0.026	±	0.005	0.359	±	0.015	***	0.730	±	0.070	***
PC(39:5)	0.021	±	0.005	0.156	±	0.005	***	0.291	±	0.025	***
PC(41:7)	0.001	±	0.001	0.008	±	0.002	*	0.020	±	0.001	***
PC(41:6)	0.001	±	0.000	0.073	±	0.004	***	0.139	±	0.016	***
PC(41:5)	0.002	±	0.001	0.012	±	0.001	**	0.034	±	0.003	***
PC-O(37:6)	0.006	±	0.001	0.101	±	0.014	**	0.140	±	0.023	***
PC-O(37:5)	0.027	±	0.007	0.211	±	0.024	*	0.311	±	0.066	**
PC-O(39:6)	0.009	±	0.004	0.108	±	0.009	**	0.130	±	0.023	***
PC-O(39:5)	0.002	±	0.001	0.072	±	0.012	NS	0.129	±	0.031	**
PC-O(41:6)	0.000	±	0.000	0.007	±	0.002	NS	0.015	±	0.006	*
PC-O(41:5)	0.000	±	0.000	0.003	±	0.001	NS	0.012	±	0.005	NS
PE(37:6)	0.000	±	0.000	0.014	±	0.001	**	0.031	±	0.004	***
PE(37:5)	0.001	±	0.000	0.069	±	0.008	**	0.144	±	0.016	***
PE(39:7)	0.001	±	0.000	0.005	±	0.001	**	0.011	±	0.001	***
PE(39:6)	0.029	±	0.007	0.145	±	0.012	***	0.237	±	0.019	***
PE(39:5)	0.008	±	0.004	0.164	±	0.016	**	0.302	±	0.035	***
PE(41:6)	0.002	±	0.001	0.023	±	0.002	***	0.040	±	0.002	***
PE-P(37:5)	0.023	±	0.002	0.459	±	0.073	***	0.517	±	0.062	***
PE-P(39:6)	0.002	±	0.002	0.143	±	0.011	***	0.139	±	0.007	***
PE-P(39:5)	0.034	±	0.004	0.263	±	0.031	***	0.329	±	0.036	***
PE-P(41:5)	0.004	±	0.001	0.033	±	0.004	***	0.044	±	0.005	***
LPI(21:5)	0.000	±	0.000	0.000	±	0.000	NS	0.001	±	0.000	NS
PI(37:6)	0.000	±	0.000	0.002	±	0.000	**	0.007	±	0.000	***
PI(37:5)	0.006	±	0.001	0.067	±	0.004	***	0.105	±	0.008	***
PI(39:6)	0.003	±	0.001	0.083	±	0.006	***	0.131	±	0.007	***
PI(39:5)	0.012	±	0.002	0.281	±	0.037	**	0.455	±	0.067	***
PI(41:7)	0.000	±	0.000	0.000	±	0.000	NS	0.002	±	0.001	*
PI(41:6)	0.000	±	0.000	0.016	±	0.001	***	0.028	±	0.002	***
PI(41:5)	0.000	±	0.000	0.022	±	0.002	**	0.039	±	0.005	***
PS(37:5)	0.000	±	0.000	0.003	±	0.002	NS	0.044	±	0.004	***
PS(39:6)	0.000	±	0.000	0.029	±	0.004	**	0.048	±	0.007	***
PS(39:5)	0.000	±	0.000	0.208	±	0.015	***	0.344	±	0.033	***
PS(41:6)	0.002	±	0.001	0.021	±	0.009	NS	0.024	±	0.008	NS
PS(41:5)	0.000	±	0.000	0.010	±	0.004	NS	0.034	±	0.007	**
PG(37:6)	0.000	±	0.000	0.001	±	0.000	*	0.003	±	0.000	***
PG(37:5)	0.000	±	0.000	0.001	±	0.000	NS	0.002	±	0.000	***
PG(39:6)	0.000	±	0.000	0.003	±	0.001	*	0.006	±	0.001	***
PA(37:5)	0.000	±	0.000	0.001	±	0.000	NS	0.003	±	0.001	**
PA(39:5)	0.000	±	0.000	0.000	±	0.000	NS	0.001	±	0.000	*

Supplementary table 1. ESI-MS characterization of HPA-containing lipid species in DHA-H-treated HEK293T cells cultures (*continued*).

	Control			DHA-H (30 µM, 24h)				DHA-H (100 µM, 24h)			
**Lipid species[2]**	**Mean[1]**	**±**	**SEM[1]**	**Mean[1]**	**±**	**SEM[1]**		**Mean[1]**	**±**	**SEM[1]**	
CL(69:7)	0.000	±	0.000	0.001	±	0.000	*	0.002	±	0.000	***
CL(71:8)	0.001	±	0.000	0.004	±	0.001	**	0.008	±	0.001	***
CL(73:8)	0.001	±	0.000	0.003	±	0.000	*	0.006	±	0.001	***
DG(37:6)	0.000	±	0.000	0.000	±	0.000	NS	0.002	±	0.000	***
DG(37:5)	0.000	±	0.000	0.021	±	0.002	NS	0.105	±	0.011	***
DG(39:6)	0.000	±	0.000	0.002	±	0.001	NS	0.016	±	0.004	**
DG(39:5)	0.001	±	0.001	0.010	±	0.003	NS	0.037	±	0.010	**
TG(55:6)	0.000	±	0.000	0.002	±	0.001	NS	0.031	±	0.006	***
TG(57:7)	0.000	±	0.000	0.000	±	0.000	NS	0.009	±	0.003	*
TG(57:6)	0.000	±	0.000	0.000	±	0.000	NS	0.005	±	0.003	NS
CE(21:5)	0.000	±	0.000	0.004	±	0.002	NS	0.030	±	0.010	*
HPA (linked)[3]	**0.249**	**±**	**0.049**	**3.996**	**±**	**0.231**	***	**7.032**	**±**	**0.711**	***
HPA (free)[4]	**0.000**	**±**	**0.000**	**0.001**	**±**	**0.000**	**NS**	**0.005**	**±**	**0.001**	**

[1]Lipid species are expressed as mol% of total membrane lipids (mean ± SEM). All data was normalized accordingly with the protein content in samples. [2]The sum formula, e.g., PC(37:5) specifies first the total number of carbons in the fatty acid legs and then the sum of double bonds. [3]HPA (linked) make reference to total lipids containing HPA in their structures. [4]HPA (free) make reference to the free fatty acid forms. ANOVA & Tukey's post hoc test: NS: Not Significant; * p<0.05, ** p<0.01 and *** p<0.001 as compared with control condition.

Supplementary table 2. ESI-MS characterization of DHA-H-containing lipid species in DHA-H-treated HEK293T cells cultures.

	Control			DHA-H (30 µM, 24h)				DHA-H (100 µM, 24h)			
**Lipid species[2]**	**Mean[1]**	**±**	**SEM[1]**	**Mean[1]**	**±**	**SEM[1]**		**Mean[1]**	**±**	**SEM[1]**	
PC(36:6:1)	0.000	±	0.000	0.004	±	0.001	NS	0.015	±	0.002	***
PC(38:7:1)	0.000	±	0.000	0.016	±	0.002	***	0.044	±	0.002	***
PC(38:6:1)	0.000	±	0.000	0.109	±	0.009	***	0.280	±	0.009	***
PC(40:7:1)	0.000	±	0.000	0.094	±	0.006	***	0.234	±	0.004	***
PC(40:6:1)	0.000	±	0.000	0.024	±	0.003	**	0.075	±	0.006	***
PE(38:7:1)	0.000	±	0.000	0.006	±	0.001	NS	0.009	±	0.002	**
PE(38:6:1)	0.000	±	0.000	0.029	±	0.002	***	0.071	±	0.004	***
PE(40:7:1)	0.000	±	0.000	0.042	±	0.003	***	0.096	±	0.006	***
PE(40:6:1)	0.000	±	0.000	0.056	±	0.003	***	0.145	±	0.007	***
PE(42:7:1)	0.000	±	0.000	0.002	±	0.001	NS	0.009	±	0.002	***
PI(38:6:1)	0.000	±	0.000	0.012	±	0.001	***	0.019	±	0.001	***
PI(40:6:1)	0.000	±	0.000	0.014	±	0.001	***	0.025	±	0.002	***
PS(40:7:1)	0.000	±	0.000	0.003	±	0.001	*	0.007	±	0.001	***
PS(40:6:1)	0.000	±	0.000	0.024	±	0.002	***	0.066	±	0.004	***
PA(38:6:1)	0.000	±	0.000	0.000	±	0.000	NS	0.002	±	0.000	***
DG(38:6:1)	0.000	±	0.000	0.003	±	0.002	NS	0.028	±	0.005	***
DHA-H (linked)[3]	**0.000**	**±**	**0.000**	**0.439**	**±**	**0.018**	***	**1.124**	**±**	**0.037**	***
DHA-H (free)[4]	**0.000**	**±**	**0.000**	**0.047**	**±**	**0.007**	**NS**	**0.268**	**±**	**0.056**	**

[1]Lipid classes are expressed as mol% of total membrane lipids (mean ± SEM). All data was normalized accordingly with the protein content in samples. [2]The sum formula, e.g., PC(38:6:1) specifies first the total number of carbons in the fatty acid legs then the sum of double bonds, while the third number indicates the hydroxyl group. [3]DHA-H (linked) make reference to total lipids containing DHA-H in their structures. [4]DHA-H (free) make reference to the free fatty acid forms. ANOVA & Tukey's post hoc test: NS: Not Significant; * p<0.05, ** p<0.01 and *** p<0.001 as compared with control condition
